# Letter to the Editor: Type IV Is Not Type IV

**DOI:** 10.5435/JAAOSGlobal-D-21-00313

**Published:** 2022-03-02

**Authors:** Thomas W. Bauer

**Affiliations:** From the Pathologist-in-Chief & Director, Professor of Pathology and Laboratory Medicine, Weill Cornell Medical College, Hospital for Special Surgery, New York, NY.

**To the Editor:** This concerns important misconceptions inherent in a review article entitled: “Metal hypersensitivity in joint arthroplasty” by Johannes Michiel van der Merwe (*JAAOS Glob Res Rev* 2021;5:1-8).^1^

Different types of hypersensitivity reactions are often classified according to the Gell-Coombs Classification^2^ published in 1963. Although far from perfect, this commonly used classification is a good starting point for discussing the pathogenesis, symptoms, pathology, and treatment of various types of hypersensitivity reactions (Table 1). Histologic findings in tissue associated with a hypersensitivity reaction often reflect the underlying pathophysiology. For example, a mucosal biopsy associated with a type I reaction is likely to show edema, mast cells, and eosinophils. In type II hypersensitivity, IgG and IgM may cause cell lysis or induce subsequent phagocytosis of affected cells by macrophages without inflammation. Type IV hypersensitivity involves lymphocytes and macrophages and may demonstrate granulomas. The adaptive immune reaction that some patients develop in response to metal ions or particles from articular surfaces or modular connections is thought to represent a type IV hypersensitivity reaction.

**Table 1:** Gell-Coombs Classification of Hypersensitivity Reactions (Modified From Reference #2)

Type I: Immediate Hypersensitivity. A reaction that may occur within minutes of exposure to an allergen (eg, pollen, a bee sting, nuts, or shellfish). The allergen interacts with cell-bound IgE, causing degranulation of mast cells and basophils. This leads to increased vascular permeability, edema, and inflammation.
Type II: Antibody-dependent cytotoxicity. Destruction of cells by preexisting immunoglobulins in a sensitized individual. For example, a transfusion reaction in which circulating IgG and IgM interact with antigens on the surface of transfused blood cells or a drug reaction in which immunoglobulins interpret membrane bound drug as foreign.
Type III. Immune complex-mediated hypersensitivity. Antigen-antibody complexes are deposited in tissue, often blood vessels, and activate complement causing local tissue damage.
Type IV: Cell-mediated hypersensitivity. A complex reaction (now with four subtypes) involving primarily lymphocytes. It is the expected reaction to some types of infection and to organ transplant rejection and contact hypersensitivity.

**Table 2:** Simplified Krenn Classification of Joint Implant-Related Pathology (Modified From Reference #3)

Type I: Particle Type
Type II: Infectious type
Type III: Combined type
Type IV: Indifferent type (subset with lymphocytes suggesting immunologic reaction)

Van der Merwe notes, “MH [metal hypersensitivity] is a type IV HS [hypersensitivity] reaction.” He further notes, “the difference between a type IV HS reaction and a type I or II HS reaction is that no or very small amounts of wear particles or inflammatory infiltrates are seen histologically in type IV reactions.” That statement makes no sense in the context of the Gell-Coombs Classification that we all use in the context of hypersensitivity reactions, but a review of citation 15 reveals the problem: Van der Merwe is not referring to the Gell-Coombs Classification of hypersensitivity reactions but, instead, is referring to the Krenn^3^ modification of the Morawietz^4^ classification of periprosthetic histology, which is only indirectly related to hypersensitivity (Table 2). Van der Merwe has modified the Krenn classification without adequate citations in his Figure 2, incorrectly suggesting that the Krenn classification refers to four different types of hypersensitivity reactions. In fact, Krenn type I represents an innate, macrophage reaction to particles (not a hypersensitivity reaction at all), and type II reflects periprosthetic infection. The histology of Krenn type I contains macrophages, giant cells, and debris, while Krenn type II contains neutrophils, but this is not true of Gell-Coombs Types I and II. Van der Merwe further confuses readers by attempting to merge the two classifications, for example, suggesting that type 2 with neutrophils represents hypersensitivity. It does not; it represents periprosthetic infection. Readers of *JAAOS Global Research & Reviews* should interpret the contents of the van der Merwe review with caution.
